# Resilience Optimization of Post-Quantum Cryptography Key Encapsulation Algorithms

**DOI:** 10.3390/s23125379

**Published:** 2023-06-06

**Authors:** Sana Farooq, Ayesha Altaf, Faiza Iqbal, Ernesto Bautista Thompson, Debora Libertad Ramírez Vargas, Isabel de la Torre Díez, Imran Ashraf

**Affiliations:** 1 Department of Computer Science, University of Engineering & Technology (UET), Lahore 54890, Pakistan; sanifarooq007@gmail.com (S.F.); faiza.iqbal@uet.edu.pk (F.I.); 2 Higher Polytechnic School, Universidad Europea del Atlántico, Isabel Torres 21, 39011 Santander, Spain; ernesto.bautista@unini.edu.mx (E.B.T.); debora.ramirez@unini.edu.mx (D.L.R.V.); 3 Department of Project Management, Universidad Internacional Iberoamericana, Campeche 24560, Mexico; 4 Project Management, Universidad Internacional Iberoamericana, Arecibo, PR 00613, USA; 5 Universidade Internacional do Cuanza, Estrada Nacional 250, Bairro Kaluapanda, Cuito EN250, Angola; 6 Department of Signal Theory, Communications and Telematics Engineering, Unviersity of Valladolid, Paseo de Belén, 15, 47011 Valladolid, Spain; isator@tel.uva.es; 7 Department of Information and Communication Engineering, Yeungnam University, Gyeongsan 38541, Republic of Korea

**Keywords:** cryptography, post-quantum cryptography, asymmetric cryptography, key encapsulation mechanism, BIKE, classic McEliece

## Abstract

Recent developments in quantum computing have shed light on the shortcomings of the conventional public cryptosystem. Even while Shor’s algorithm cannot yet be implemented on quantum computers, it indicates that asymmetric key encryption will not be practicable or secure in the near future. The National Institute of Standards and Technology (NIST) has started looking for a post-quantum encryption algorithm that is resistant to the development of future quantum computers as a response to this security concern. The current focus is on standardizing asymmetric cryptography that should be impenetrable by a quantum computer. This has become increasingly important in recent years. Currently, the process of standardizing asymmetric cryptography is coming very close to being finished. This study evaluated the performance of two post-quantum cryptography (PQC) algorithms, both of which were selected as NIST fourth-round finalists. The research assessed the key generation, encapsulation, and decapsulation operations, providing insights into their efficiency and suitability for real-world applications. Further research and standardization efforts are required to enable secure and efficient post-quantum encryption. When selecting appropriate post-quantum encryption algorithms for specific applications, factors such as security levels, performance requirements, key sizes, and platform compatibility should be taken into account. This paper provides helpful insight for post-quantum cryptography researchers and practitioners, assisting in the decision-making process for selecting appropriate algorithms to protect confidential data in the age of quantum computing.

## 1. Introduction

Cryptography is a method of protecting data in the presence of unauthorized users by establishing a secure communication channel between two parties. This technique was developed by the Institute of Electrical and Electronics Engineers (IEEE). Encryption and decryption are the two processes that are carried out in cryptography by both the sender and the receiver. The process of converting unencoded data into an encoded format referred to as a “Cipher” using a safe data source is what we mean when we talk about encryption (key). Decryption is the process of converting encrypted data back into its original plain form using either the same secure data source (key) or a different secure data source [1]. This process is the inverse of encryption.

**Figure 1:**
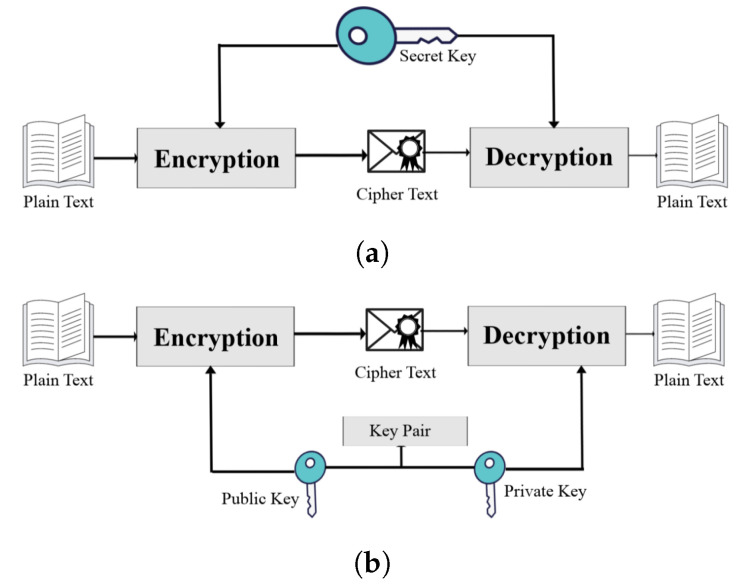
Types of cryptography, (**a**) symmetric cryptography, and (**b**) asymmetric cryptography.

There are two distinct categories of cryptographic methods: symmetric and asymmetric. The process of the encryption and decryption of data in symmetric cryptography only requires the use of a single key. A private key is utilized to carry out this method. This refers to the requirement that a private key must be guarded in secrecy and given out to a sender and recipient who are authorized to do so. The process of symmetric cryptography is illustrated in Figure 1a. For encryption and decryption, asymmetric cryptography, also known as public key cryptography, employs a key pair. One of the keys in the key pair is a publicly accessible key. The sender will use a public key for encryption, while the recipient will use the private key, which is only known to them [2]. Figure 1b depicts the operation of an asymmetrical cryptography system.

The use of quantum computers, with their unfathomable computing power, is rapidly approaching reality [3]; it is no longer a dream. A computer based on the peculiar properties of quantum mechanics can perform calculations exponentially faster than a computer made of classical bits. In October 2019, Google announced the development of a quantum computer that samples the output of a pseudo-random quantum circuit ten-times faster than the fastest supercomputers available today [4]. Recent developments in quantum computing pose a threat to public key primitives [5] due to quantum computers’ ability to solve complex cryptographic problems in polynomial time. post-quantum cryptography (PQC) refers to asymmetric cryptographic algorithms that can withstand attacks from a quantum computer.

The National Institute of Standards and Technology (NIST) is currently developing a new generation of quantum-resistant key encapsulation and authentication schemes [6] to combat this threat to essential Internet security protocols such as transport layer security (TLS). TLS [7] is the most-popularly employed secure communication protocol for online page transfers, encrypted email server access, and mobile applications. The majority of hypertext transfer protocol (HTTPS) service connections utilize TLS [8]. TLS uses Rivest–Shamir–Adleman (RSA) or elliptic curve (EC) signatures and Diffie–Hellman (DH) with EC for key exchange. It is crucial to plan the transition to quantum-resistant schemes, such as Secure Hashing Algorithm-2 (SHA-2) and elliptic curve digital signature algorithm (EC), which were not adopted until a decade after their standardization [9,10], given that the adoption of cryptographic techniques could take years. It is important to note that increasing the hash size of SHA-2 does not give essential protection against quantum assaults. Larger hash sizes can provide temporary mitigation against some attacks, but they are not an adequate remedy [11]. Quantum computers can still potentially break cryptographic schemes based on hash functions by using algorithms specifically designed for quantum computing. It is necessary to adopt algorithms and protocols that are designed explicitly to survive the assaults of quantum computers. These methods typically rely on mathematical problems that both classical and quantum computers both find hard to solve. We may already be investigating the impact of PQC on real-world performance, whereas NIST takes performance, security, and other factors into account when selecting algorithms for standardization. Therefore, the performance of the TLS handshake is of great importance [12,13].

Digital signatures and key exchange algorithms are required for two parties to generate a shared key and verify its authenticity. Signature algorithms (SIG) are utilized for sender authentication, while key encapsulation algorithms (KEM) are utilized for key exchange. In contrast to previous research, which typically only examined NIST third-round finalist PQC algorithms or a limited selection of methods, we tested the performance advantages of various combinations of NIST fourth-round finalist PQC algorithms for the two widely used operating system (OS)s Windows and Linux. This allowed us to not only conclude how PQC will impact day-to-day usage, but also compare the performance of the PQC algorithm to that of the NIST third- and fourth-round finalists [14,15].

The rest of the study is organized as follows. Related work is presented in Section 2 along with the motivation behind PQC, highlighting the limitations of classical cryptographic algorithms and the need for PQC. Section 3 provides a brief overview of the methodology used in this study to evaluate PQC algorithms, including classic McEliece and BIKE. Section 4 discusses the experimental results, which include performance metrics such as encapsulation and encapsulation times. Finally, in Section 5, our conclusions and recommendations for future research are given.

## 2. Related Work

As the power of quantum computers increases in the foreseeable future, we must consider how this will affect Internet security. Given the power of quantum computing, significant research effort is being devoted to solving the difficult problems used in modern cryptography, which is expected to have a significant impact on the security of current classic public key cryptosystems in the near future.

PQC refers to asymmetric cryptographic methods that are immune to quantum-computer-based attacks. Shor’s algorithm was one of the first to demonstrate that three problems that serve as the foundation of classical public key cryptography can be solved in polynomial time by a quantum computer’s exponential computing power. Shor’s algorithm, which has the potential to break a number into prime factors in polynomial time, poses a threat to all currently popular asymmetric algorithms based on the integer factorization problem, such as RSA, the discrete logarithm problem, elliptic curve cryptography (ECC), and elliptic curve discrete logarithm (ECDH) [16]. Even if there are no quantum computers capable of running Shor’s algorithm on a reasonably sized asymmetric key today, one will exist in the future [17].

Due to the aforementioned circumstances, it is becoming increasingly important to design a new quantum-safe encryption and authentication system that is not based on the difficult problems of classical public key cryptography. TLS’s handshake protocol heavily depends on variants of RSA, DH, and EC for signing and key exchange. In order to be resistant to quantum computers, these algorithms must be replaced with PQC algorithms. Several post-quantum cryptography alternatives have been proposed. The current five families of PQC systems are code-based, lattice-based, hash-based, multivariate, and supersingular elliptic curve isogeny cryptography. The most-developed digital signature methods are hash-based. They were presented for the first time in 1979 by Lamport [18], Merkle, and Winternitz and have since undergone significant enhancements [19]. They provide a high level of security and have been evaluated thoroughly. Ajtai introduced lattice-based cryptography for the first time in 1996 [15]. In comparison to other PQC families, it offers highly effective key encapsulation techniques. In contrast to hash-based techniques, however, their security is less well known.

KEM and SIG algorithms are both required for establishing a shared key and verifying the authentication of two parties. Similar to previous NIST efforts to standardize various sub-fields of cryptography, most notably the 2001 standardization of AES, NIST is currently engaged in a project to standardize PQC algorithms. Numerous research analyses on the PQC algorithms and their alternatives that have been presented and advanced to the third round of the NIST standardization competition have already been conducted. Two additional classifications exist for algorithms. The KEM algorithm is used for key exchange, while the SIG algorithm is used for sender authentication. The key encapsulation mechanisms and SIG algorithms [15,20] are displayed in Table 1.

Past research has measured the performance of NIST third-round finalist PQC algorithms in TLS handshakes based on a variety of parameters and NIST-established security levels. NIST has standardized five levels of security strength, with Level 1 being the least-secure and Level 5 being the most-secure [20].

Table 2 compares security levels to the difficulty of breaking classical encryption or hashing algorithms with suitable key lengths. Table 3 and Table 4 display related work on PQC algorithms and signatures for performance evaluation.

NIST has already selected four third-round candidates for standardization and four for further review and research in the fourth round [24]. Table 5 and Table 6 detail these algorithms.

In the era of the Internet of Things (IoT), NIST is concurrently conducting the Lightweight Cryptography Standardization Process (LWC-SP), which aims to select lightweight cryptographic algorithms for standardization. This process ensures that the chosen algorithms are secure, efficient, and suitable for resource-constrained devices. Extensive research has been conducted over the years on various lightweight cryptographic algorithms, studying their principles, techniques, and countermeasures against fault attacks. These studies provide valuable insights into the vulnerabilities and propose effective mitigation strategies in the context of lightweight cryptography. Prominent algorithms in this field include Pomaranch Cipher [25], Grostl Hash, Midori Cipher [26], RECTANGLE Cipher [27], and Ascon [28]. Notably, in the latest updates in February 2023, NIST has finalized the standardization of the Ascon algorithm as part of the LWC-SP. The updates from NIST affirm that Ascon is a secure and efficient lightweight block cipher. Ascon demonstrates versatility in implementation across different platforms and exhibits resistance against various attack vectors. Given these qualities, Ascon emerges as a favorable option for deployment in resource-constrained devices [29]. Evaluating the PQC algorithms for lightweight cryptography or embedded systems is of utmost importance. Therefore, researchers have been studying and conducting evaluations of PQC algorithms on ARM Cortex M4 processors to establish benchmarks [23].

Not every combination of PQC signature and key exchange algorithms could be considered for this study. In light of this, we chose two of the PQC algorithms, bit-flipping key encapsulation (BIKE) and classic McEliece, for performance evaluation in this study, which are fourth-round NIST finalists. Unlike prior studies, which focused on PQC algorithms from a single family [30], this research took a broader approach by evaluating and comparing all possible variants of PQC algorithms under scrutiny. This approach went beyond the historical context and delves into the evaluation and performance analysis of all variants, NIST PQC forth-round finalists, and alternate candidates on two different operating systems. We examined the key lengths, private key lengths, ciphertext lengths, and key exchange methods of these algorithms to offer a full study of their practicality and applicability for real-world applications. For key encapsulation, BIKE is a code-based algorithm. BIKE’s security depends on a challenging problem in coding theory [31]. Due to the recent update to the implementation, we cannot comment on the security of BIKE on multiple fronts. However, NIST considers it a promising candidate and has advanced it to the final round [24]. Once all security concerns have been addressed, standardization may be considered.

Classic McEliece is the oldest cryptosystem proposed in Round 4 of the NIST PQC standard [24] submissions. Based on the 1979 McEliece cryptosystem that employed secret Goppa codes, the original cryptosystem was not designed to adhere to restrictions on public use computation. Researchers in the field of cryptography have thoroughly examined and analyzed the McEliece cryptosystem during the course of its history. Numerous assaults have been considered and planned, but none have been able to undermine the scheme’s entirety. The following notable assaults have been investigated:

- Information set decoding (ISD) attack:This attack served as the foundation for the initial assault plan against McEliece. This approach attempted to discover a small set of linearly dependent syndromes in order to retrieve the private key. It was later demonstrated, though, that, with appropriate parameter selection, this attack is not possible.
- Square root attack: This attack was introduced in the context of McEliece variants, such as the Niederreiter cryptosystem. This attack tries to recover the private key by taking advantage of the algebraic structure of the cryptosystem’s code. This attack, however, is only relevant to certain parameter selections and is not seen as being practical against versions of McEliece that have been properly configured [32].
- Meet-in-the-middle attack: This attack tries to exploit the error-correction capability of the code and the encoding process to retrieve the private key. This assault, however, needs an excessive amount of processing power and is not seen as a viable threat.

Classic McEliece provides security against chosen ciphertext attack (CCA). When the message is selected at random, an attacker cannot decipher the message efficiently using the cipher text and public key. The original cryptosystem offered security against one-way CCA, meaning an attacker cannot efficiently decipher a message from a ciphertext and a public key when the message is chosen at random [33]. This system combines NTS-KEM and classic McEliece in order to provide efficient implementation and CCA security. It implements the hashing of errors added to the cipher text and relies on the security provided by hash functions. It is a defense against CCA attacks.

Although the performance comparison of the BIKE and classic McEliece algorithms was the main emphasis of this work, it is important to be aware of various sorts of attacks so that practical defenses against these threats can be evaluated in future research. The side-channel attacks (SCAs) can exploit information leaked through the physical characteristics of the cryptographic implementation. Two common types of SCAs are fault attacks and power analysis attacks.

Fault attacks include introducing faults or errors within the cryptographic system on purpose to obtain unauthorized access or extract sensitive information. An attacker can compel the system to act unexpectedly by changing the execution environment, potentially disclosing private keys or other confidential data. Fault attacks can be a serious threat to the security of cryptographic devices. To preserve the integrity and security of cryptographic operations, countermeasures against fault attacks include techniques such as redundancy, error detection, error correction, and fault-tolerant-designed cryptography devices [34]. In contrast, power analysis attacks seek to leverage power consumption patterns or electromagnetic radiation released during cryptographic operations. An attacker can learn about the secret keys or intermediate values utilized in the calculations by analyzing these side-channel signals. Techniques such as power-analysis-resistant designs, randomizing power usage, and implementing secure masking systems are examples of countermeasures against power analysis attacks [35].

In combined attacks, adversaries combine numerous attack strategies to maximize their chances of success. A combined fault and power analysis attack, for example, may include generating faults in the system while concurrently monitoring power usage to obtain sensitive information. Assessing and managing the risks associated with combination assaults requires a thorough examination of the system’s resistance to both fault and power analysis attacks.

While this study work gives useful insights into the performance details of the Classic McEliece and BIKE algorithms, more research is needed to fully analyze the resilience of PQC algorithms against the active and passive side-channel attacks, as well as their associated countermeasures.

## 3. Proposed Methodology

**Figure 2:**
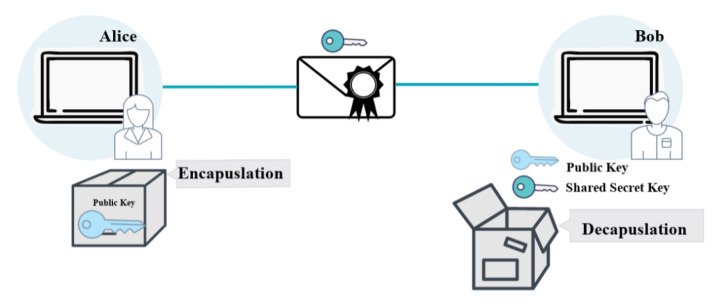
Key encapsulation mechanism overview.

**Figure 3:**
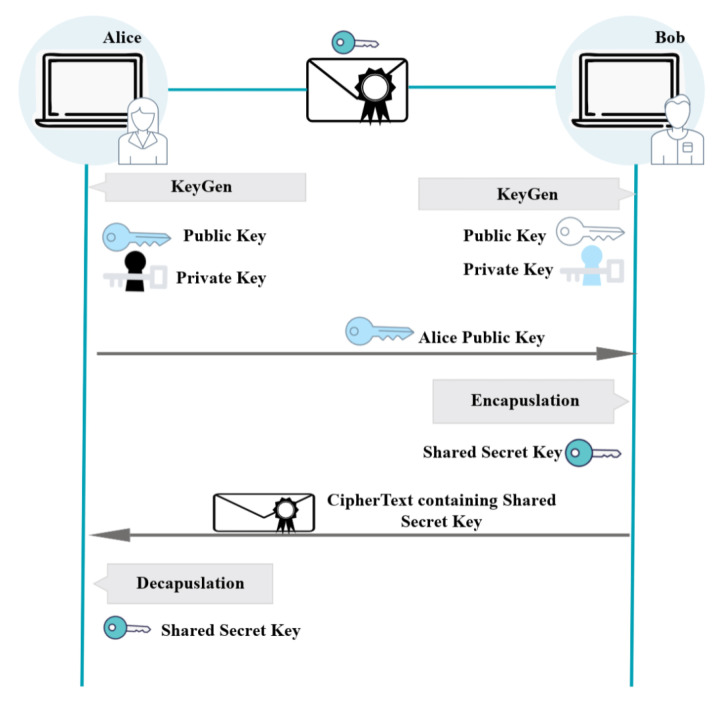
Key encapsulation mechanism detail.

KEM is a cryptographic technique used to transmit a secret key over an unsecured communication channel. The secret key is encapsulated in a layer of encryption before being transmitted to the intended recipient, as displayed in Figure 2 and Figure 3. After receiving the encapsulated key, the recipient can use a separate key, known as a “key-decapsulation key”, to decrypt and recover the original secret key. This allows for secure key exchange without the need for a shared secret key to be established beforehand. Key generation and encapsulation/decapsulation are the two main steps of a KEM process.

In the key-generation step, one of the entities generates a shared secret key. The secret key is then encapsulated, or surrounded, by a collection of public parameters. The encapsulated key is then transmitted to the other entity, typically referred to as the “user” or “receiver”. The recipient decapsulates the key using its own private key, thereby revealing the shared secret key. Encapsulation and decapsulation typically involve the use of mathematical algorithms. KEMs are widely used in a variety of cryptographic systems to exchange keys securely. Typical applications of KEMs include:

- Secure communication:KEMs can be utilized to generate a shared secret key between two parties, which can then be used to encrypt and decrypt communications.
- Key exchange: KEMs can be utilized to securely exchange a secret key between two entities, thereby enabling the establishment of a secure communication channel.
- Key agreement: Multiple entities can establish a secure communication channel by using KEMs to establish a shared secret key.
- Key derivation: KEMs can be used to generate a secret key from a master key, which can then be used for cryptographic operations such as encryption and signing.
- Authentication: A client and a server can use KEMs as part of an authentication scheme to establish a shared secret key, which can then be used to authenticate the client [36]. This shared secret key can then be used to authenticate the client.
- Hybrid encryption: KEMs can be utilized to encrypt a symmetric key. A large amount of data can be encrypted using the symmetric encryption key, while the KEM key can be used to encrypt the symmetric encryption key.
- Post-quantum cryptography: KEMs are also utilized in post-quantum cryptography, which aims to protect against possible quantum computing attacks.

Several metrics can be used to evaluate the performance of a KEM, such as:

- Key size: the size of the KEM-generated shared secret key. In general, a smaller key size is considered to be more secure and efficient.
- Computational cost: Key generation, encapsulation, and decapsulation demand a certain amount of computational resources. In general, a lower computational cost is regarded as more efficient.
- Communication cost: The quantity of information that must be transmitted during encapsulation and decapsulation. In general, a lower communication cost is considered more efficient.
- Security: The level of security provided by the KEM is typically measured in terms of the number of required operations to compromise the system. In general, a higher level of security is considered to be more secure.
- Error rate: the frequency of errors that occur during key generation or encryption/decryption. A lower error rate is generally regarded as more trustworthy.
- Time expense: time required for the key generation, encapsulation, and decapsulation operations. In general, a lower time cost is considered more efficient.

In this paper, we present our proposed methodology for measuring and comparing the performance of different KEMs using the liboqs [37]. Our methodology aims to provide a comprehensive evaluation of KEM algorithms, considering metrics such as security level, computational cost, and time cost. The motivation behind developing our proposed methodology resulted from a necessity for a standardized and reliable approach to evaluate KEM algorithms. The evaluation based on a comprehensive set of evaluation metrics considered real-world performance scenarios on the two most-widely used operating systems. Our methodology aimed to provide an adequate foundation for evaluating the effectiveness of KEMs and making appropriate decisions.

Our proposed methodology offers several unique features. These include:

- Comprehensive evaluation metrics: We considered a range of evaluation metrics, including key size, computational cost, security level, error rate, and time expense. This comprehensive set of metrics allows for a holistic assessment of KEM algorithms and enables researchers to understand their performance characteristics from multiple perspectives.
- Improved measurement accuracy: We incorporated optimizations in the measurement process to enhance accuracy and consistency. By leveraging the liboqs [37] library, we ensured a standardized implementation and reliable measurement results across different KEM algorithms.

The following methodology was used to measure the performance of a KEM using the liboqs [37]:

- Initialization and configuration: The OQS library was initialized, and the parameters for the KEM algorithm were set. This included specifying the security level, which determined the key size and strength of security guarantees.
- Setting up the loop: A loop was set up to perform multiple iterations of the KEM algorithm, in order to obtain the average time for the performance.
- Key generation: Inside the loop, a random private key and corresponding public key were generated for the KEM algorithm being tested.
- Timing key pair generation: The time taken to generate the key pair was measured using the CPU clock. The method was run using the previously generated keys, and the resulting shared secret was discarded.
- Calculating average time for key pair generation: After the method was run for the desired number of iterations, the average time taken for the key pair generation was calculated.
- Timing KEM encapsulation: The time taken to perform the KEM encapsulation was measured using the CPU clock. The method was run using the previously generated public key and a secret message.
- Calculating average time for KEM encapsulation: After the method was run for the desired number of iterations, the average time taken for the KEM encapsulation was calculated.
- Timing KEM decapsulation: The time taken to perform the KEM decapsulation was measured using the CPU clock. The method was run using the previously generated private key and the ciphered secret message.
- Calculating average time for KEM decapsulation: After the method was run for the desired number of iterations, the average time taken for the KEM decapsulation was calculated.
- Printing relevant information: The name of the key exchange method, the security level, the average time, and the CPU clock speed were printed to the console for analysis.

The above steps provide a high-level overview of our methodology. These steps ensured that our proposed methodology captured the essential performance characteristics of KEM algorithms accurately and efficiently. We intended to investigate additional evaluation metrics in future work. We also wanted to include more diverse and realistic performance scenarios in order to properly analyze the practical usability of KEM algorithms with TLS.

**Figure 4:**
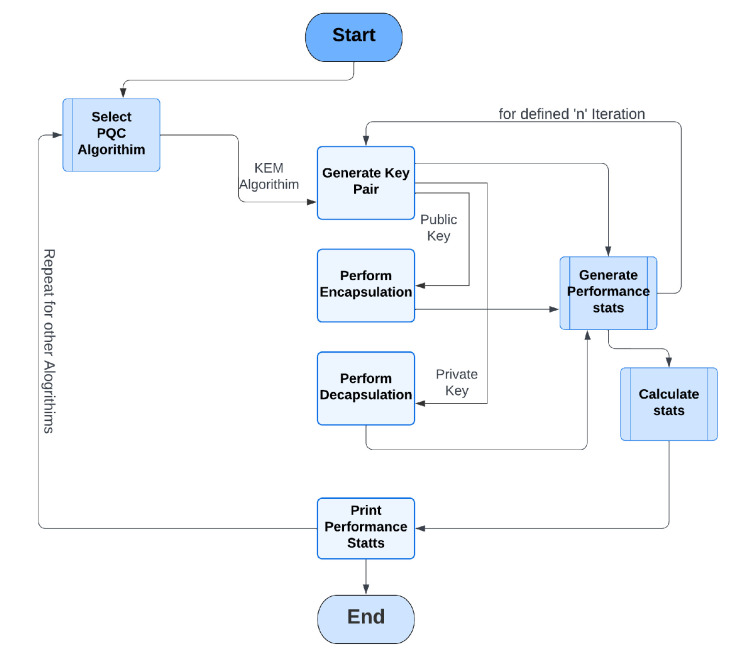
Workflow of the methodology for performance evaluation.

Overall, the methodology represented in Figure 4 was used for the measurement and comparison of the performance of different KEM algorithms using the liboqs. Algorithm 1 shows the pseudocode for the implementation of the proposed approach.**Algorithm 1:** Evaluation of PQC algorithms for Windows and Linux.
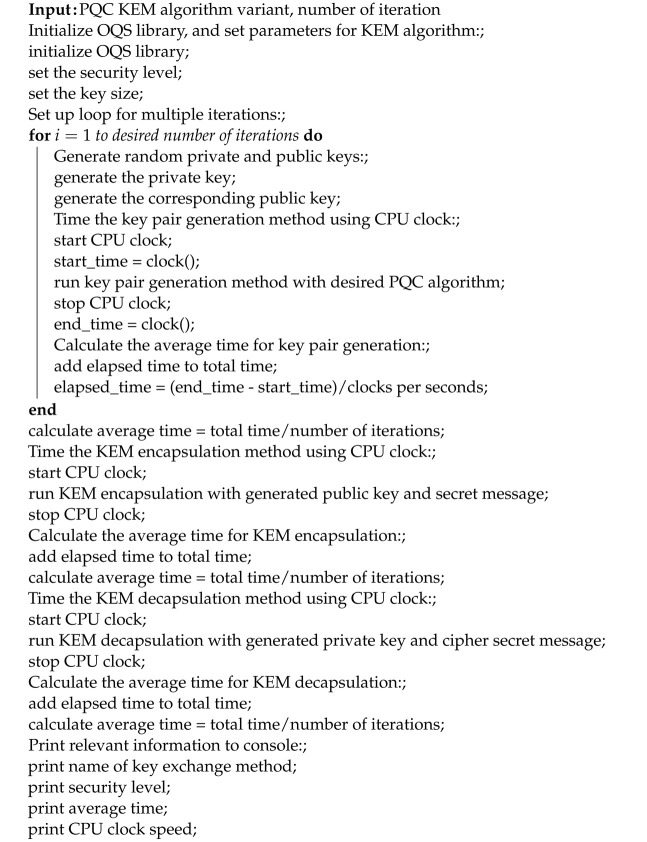


## 4. Results

### 4.1. Classic McEliece

Classic McEliece is a KEM based on the binary Goppa code. Due to the utilization of Goppa codes, the algorithm has impeccable precision. Every cipher text created with the encapsulation/encryption function can be successfully decrypted with the decapsulation/decryption function in a completely correct KEM or PKE.

In situations where a public key is frequently reused and does not need to be retransmitted for each new communication, the classic McEliece’s performance profile may be advantageous. Classic McEliece has the smallest cipher text sizes of all NIST PQC candidates. The liboqs [37] is utilized for evaluating the Classic McEliece OQS parameter. The parameters and variants of the classic McEliece algorithm are displayed in Table 7.

### 4.2. Bit-Flipping Key Encapsulation

BIKE is a code-based KEM designed to be secure against both classical and quantum computers. BIKE’s security is based on the difficulty of locating isogenies between elliptic curves, which is believed to be challenging for both classical and quantum computers. It is intended to be both efficient and secure, with low communication overhead and robust security proof. BIKE is also intended to be adaptable, with the capacity to support a wide variety of key sizes and security levels.

BIKE is one of several post-quantum key exchange algorithms proposed to secure communication against the threat posed by quantum computers. It is undergoing standardization by NIST as part of the selection process for a new suite of post-quantum cryptographic algorithms for widespread use. The BIKE algorithm’s parameters are utilized using the OQS liboqs [37]. The BIKE algorithm’s parameters and variants are displayed in Table 8.

### 4.3. Speed Test of KEMs

For benchmarking the PQC algorithm speed on two widely used operating systems, Linux and Windows, this study utilized OQS OpenSSL [38], which implements the new post-quantum (PQ) schemes with AVX2-optimized versions via the OQS liboqs library [37]. Table 9 displays the benchmark speed results for the Linux and Windows operating systems.

**Figure 5:**
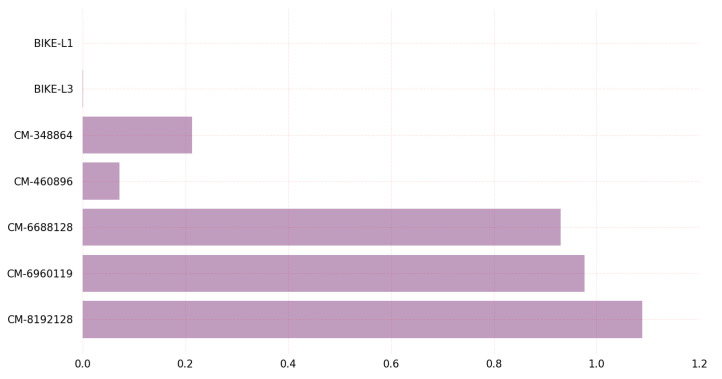
Key generation CPU clocks on Linux.

Figure 5 depicts the outcome of the KEM algorithms concerning the number of CPU cycles required for key generation. On Linux, BIKE algorithm variant BIKE-L1 claiming NIST Security Level 1 requires few CPU cycles, whereas the classic McEliece variant classic-McEliece-8192128 claiming NIST Security Level 5 requires many CPU cycles.

**Figure 6:**
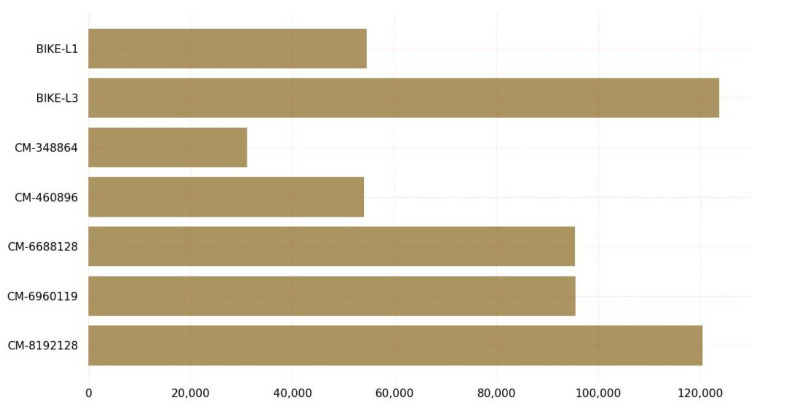
Key encapsulation CPU clocks on Linux.

The graph in Figure 5 demonstrates that, among all BIKE and classic McEliece algorithm variants, the BIKE-L1 variant requires the least amount of CPU clocks for key generation on the Linux platform. This study raised the possibility that BIKE-L1 is the most-effective BIKE algorithm version for key generation, especially in scenarios where key generation performance is a critical factor. Figure 6 depicts a comparison of KEM algorithms in terms of the CPU cycles required for encapsulation.

The graph in Figure 6 provides insights into the CPU clock requirements for key encapsulation of post-quantum cryptographic algorithms on Linux. The graph shows that the BIKE-L3 variant requires the highest number of CPU clocks for key encapsulation, while the classic McEliece variant with 128-bit security requires the least. It is worth noting that the classic McEliece and BIKE variants represent two different classes of post-quantum cryptographic algorithms. The classic McEliece is based on the McEliece cryptosystem, which relies on the hardness of decoding random linear codes. On the other hand, BIKE is a code-based PQC algorithm that is designed to provide a high level of security with relatively low key sizes.

The graph also includes key encapsulation data for other variants of the classic McEliece and BIKE algorithms. These variants offer varying levels of security and performance. For example, the classic McEliece-6960119 variant requires significantly fewer CPU clocks for key encapsulation than the BIKE-L3 variant, but provides a higher level of security. Similarly, the BIKE-L1 variant requires fewer CPU clocks for key encapsulation than the BIKE-L3 variant, but provides a lower level of security. The classic McEliece variant classic-McEliece-348864 with claimed NIST Security Level 1 requires the fewest CPU cycles for encapsulation on the Linux operating system, whereas BIKE-L3 with claimed NIST Security Level 3 requires the most CPU cycles.

**Figure 7:**
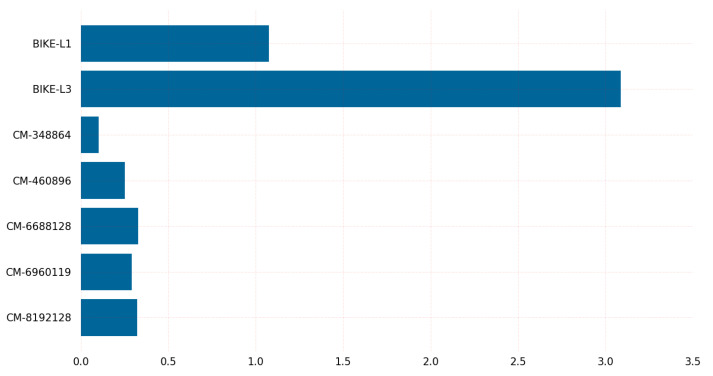
Key decapsulation CPU clocks on Linux.

Figure 7 depicts the CPU cycles required by KEM algorithms during the decapsulation procedure. Classic-McEliece-348864 required the fewest CPU cycles during the Linux decapsulation procedure, while BIKE-L3 required the most.

The graph in Figure 7 displays various variants of the PQC algorithms, including the BIKE variants and classic McEliece, and their corresponding CPU clock requirements for key decapsulation on Linux. Each variant offers a different level of security and performance and suggests that the classic McEliece variant with 128-bit security may be the most-efficient option for key decapsulation in cases where low CPU clock requirements are desirable. It provides valuable information for those seeking to select a post-quantum cryptographic algorithm for key decapsulation on Linux. The classic McEliece variant with 128-bit security may be a favorable choice for those seeking a balance between security and efficiency.

**Figure 8:**
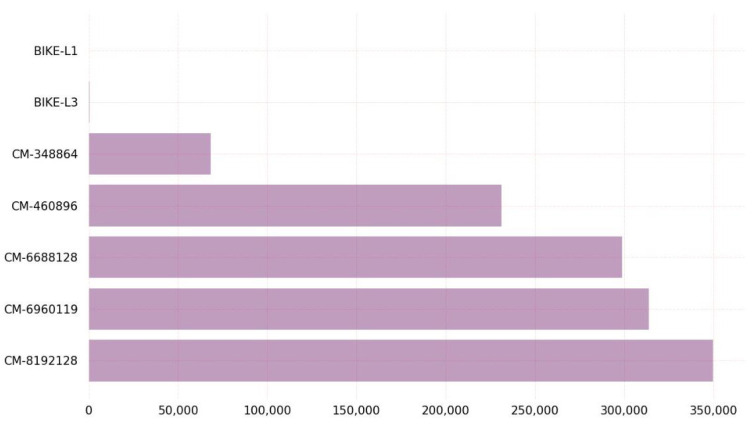
Key generation time (us) on Linux.

Figure 8 depicts the time required to generate public and private keys on Linux using the KEM algorithms. Classic-McEliece-8192128 has the largest key size in terms of bytes, as the graph clearly demonstrates. The graph in Figure 8 provides a comparison of the time required for the key generation of various variants of the BIKE and classic McEliece post-quantum cryptographic algorithms on Linux. The graph shows that the BIKE-L1 variant from the BIKE algorithm family and the classic-McEliece-348864 variant of classic McEliece require the lowest time for key generation, while the other variants require a higher amount of time. This information can be helpful for those seeking to select a post-quantum cryptographic algorithm for key generation on Linux. The BIKE-L1 and classic McEliece-348864 variants may be a favorable choice for those seeking faster key generation times, while the other variants may be suitable for those requiring higher levels of security. It is important to consider both the security and performance aspects when selecting a post-quantum cryptographic algorithm, and this graph can provide valuable insights for making an informed decision.

**Figure 9:**
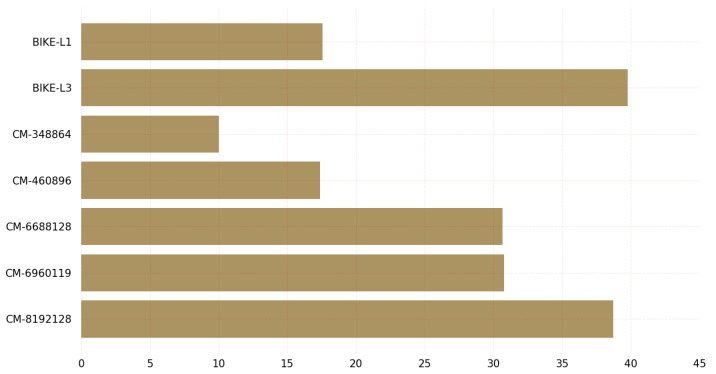
Key encapsulation time (us) on Linux.

**Figure 10:**
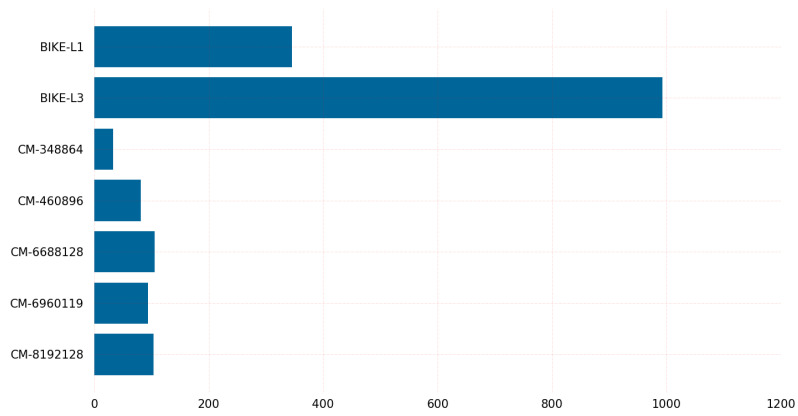
Key decapsulation time (us) on Linux.

The time cost required on Linux for encapsulation and decapsulation using PQC KEM algorithms are represented in Figure 9 and Figure 10, respectively. The graph in Figure 9 provides insights into the time required for key encapsulation of PQC algorithms on Linux. The graph shows a comparison of all variants of the BIKE and classic McEliece algorithms with respect to their time requirements for key encapsulation. The classic-McEliece-348864 variant has the lowest time requirements for key encapsulation among all variants, while the BIKE-L3 variant requires the highest amount of time. According to these results, the classic-McEliece-348864 variant may be the most-effective for key encapsulation, especially where speed is a significant consideration.

The results in Figure 10 shed light on the time required for key decapsulation of post-quantum cryptographic algorithms on Linux. The graph shows that the classic-McEliece-348864 variant has the lowest time requirements for key decapsulation among all variants of the classic McEliece algorithm, while the BIKE-L1 variant has the lowest time requirements among all variants of the BIKE algorithm. It is worth noting that the classic-McEliece-348864 variant also provides a low security level. BIKE-L3 takes more time in both the encapsulation and decapsulation processes.

**Figure 11:**
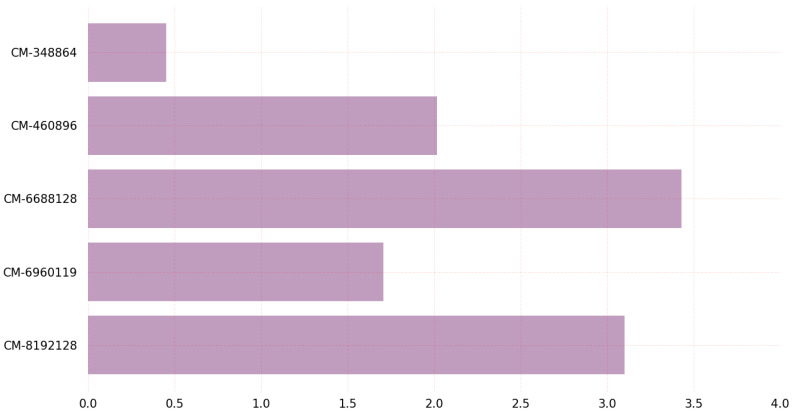
Key generation time (us) on Windows.

Figure 11 depicts the result of classic McEliece variants in terms of the time required for the key generation process on Windows. The key generation for the variant classic-McEliece-6688128 with claimed NIST Security Level 5 takes longer on Windows. The graph in Figure 11 provides insights into the time required for the key generation of classic McEliece algorithms on Windows, except for the BIKE algorithm, which is not available by default in the liboqs on Windows. The results show that the classic-McEliece-348864 variant has the lowest time requirement for key generation, followed by the classic-McEliece-6960119 variant. It is worth noting that, while the other variants have higher time requirements for key generation, they also provide higher security levels. For instance, the classic-McEliece-4608960, classic-McEliece-6688128, and classic-McEliece-8192128 variants have high security levels, but they also require higher CPU clock cycles for key generation. On the other hand, the classic-McEliece-128 and classic-McEliece-192 variants have low time requirements for key generation, but provide lower security levels.

**Figure 12:**
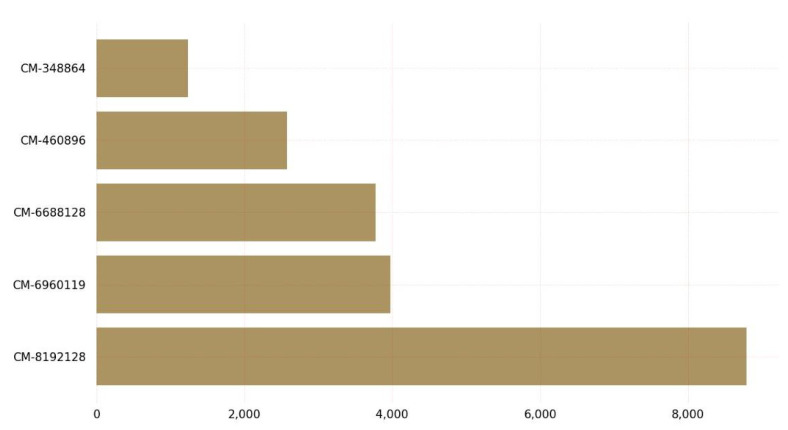
Keyencapsulation time (us) on Windows.

**Figure 13:**
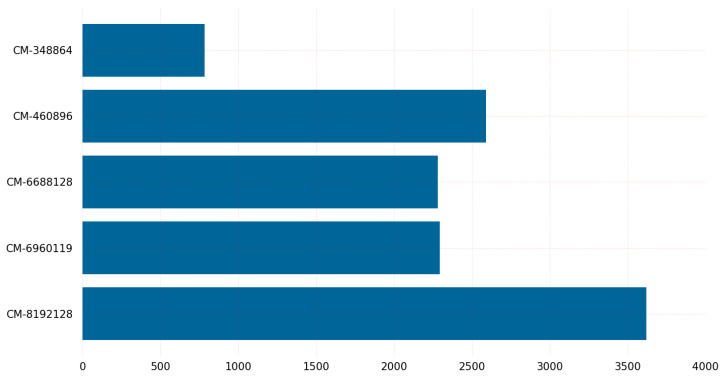
Key decapsulation time (us) on Windows.

Figure 12 and Figure 13 depict the time required on Windows for the encapsulation and decapsulation processes utilizing the PQC KEM algorithms. The classic-McEliece-8192128 algorithm requires significantly more time for both encapsulation and decapsulation. The graph in Figure 12 shows the comparison of the time required for key encapsulation on Windows between different variants of the classic McEliece PQC algorithms. The classic-McEliece-348864 variant has the lowest time requirements for key encapsulation among all variants of the classic McEliece algorithm. However, it is important to note that this variant has the lowest security level. On the other hand, the classic McEliece-8192128 variant has the highest time requirements for key encapsulation, but offers the highest level of security. Other variants such as classic McEliece-4608960, classic McEliece-6688128, and classic McEliece-6960119 have intermediate time requirements for key encapsulation and provide varying levels of security. This graph shows how important it is to carefully analyze the trade-off between efficiency and security when choosing a specific variation of a cryptographic algorithm for a specific application in the real world.

The graph in Figure 13 shows the comparison of the time required for key decapsulation on Windows between different variants of classic McEliece post-quantum cryptographic algorithms. Classic-McEliece-348864 has the least time required for key decapsulation while providing adequate security. However, the classic-McEliece-6688128 and classic-McEliece-6960119 variants showed the same time required for key decapsulation, but had different security levels. In particular, the classic-McEliece-6960119 had a higher security level than the classic-McEliece-6688128. Overall, these findings suggest that, while the classic-McEliece 348864 variant may be the most-efficient in terms of key decapsulation time, it may not provide the highest level of security. Therefore, the choice of which variant to use should depend on the specific security requirements of the system in question.

**Figure 14:**
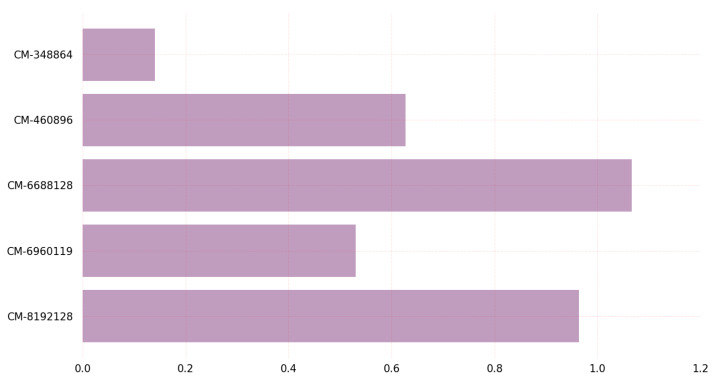
Key generation CPU clocks on Windows.

Figure 14 compares all variants of the classic McEliece PQC KEM algorithm in terms of the required CPU clocks on Windows. The KEM algorithm classic-McEliece-8192128 requires the maximum CPU cycles for encapsulation and decapsulation on Windows. The graph in Figure 14 indicates that there is a trade-off between security levels and CPU clocks required for key generation, as some classic McEliece variants with higher security levels require significantly fewer CPU clocks for key generation on Windows. This highlights the importance of choosing the appropriate post-quantum cryptographic algorithm based on specific security and performance requirements. Furthermore, it is interesting to note that the classic McEliece variants generally outperform other post-quantum cryptographic algorithms in terms of key generation efficiency, despite the potential for higher CPU clock requirements at higher security levels. This suggests that classic McEliece may be a strong candidate for practical implementation in a post-quantum secure communication system.

**Figure 15:**
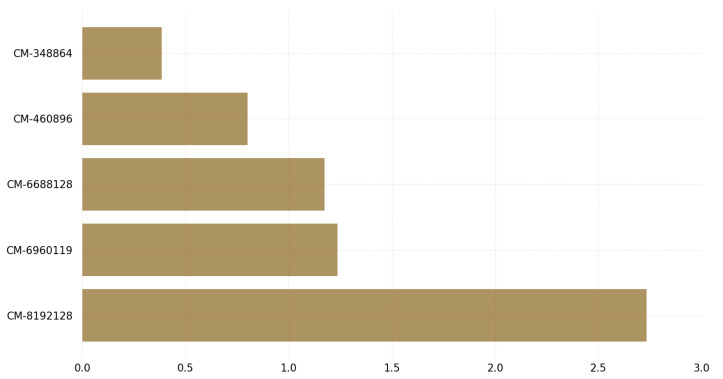
Key encapsulation CPU clocks on Windows.

The graph in Figure 15 depicts the performance of different variants of the classic McEliece PQC algorithm in terms of key encapsulation statistics with respect to CPU clocks on Windows and provides valuable insights into the practical efficiency and security of post-quantum cryptographic algorithms. Among the classic McEliece variants, the most-efficient variant for key encapsulation is the classic-McEliece-348864, which offers a 128-bit security level. Meanwhile, the classic-McEliece-6688128 requires the highest number of CPU clocks for key encapsulation. However, it is also important to note that this variant provides a very high level of security, offering a 256-bit security level, which is even higher than that of classic-McEliece-348864. By considering both the performance and security characteristics of different variants of the classic McEliece algorithm, developers and practitioners can make informed decisions about the most-appropriate post-quantum cryptographic algorithm for their needs, taking into account both speed and security requirements.

**Figure 16:**
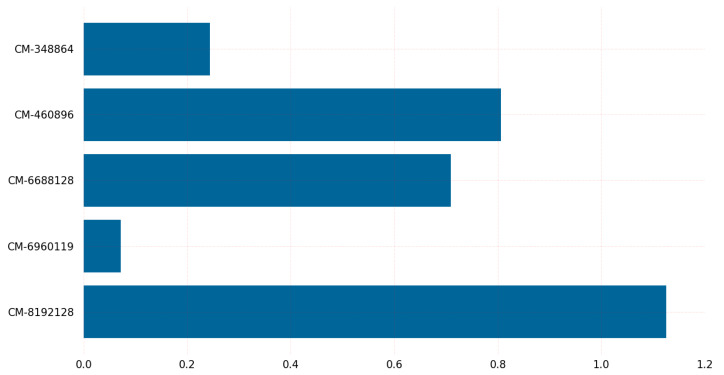
Key decapsulation CPU clocks on Windows.

The graph in Figure 16 shows the performance of different variants of the classic McEliece PQC algorithm in terms of key decapsulation statistics with respect to CPU clocks on Windows and provides valuable insights into the practical efficiency and security of post-quantum cryptographic algorithms. The data indicate that the classic McEliece-348864 is the most-efficient variant for key decapsulation on Windows, with the lowest number of CPU clocks required for this operation. The 128-bit Level 1 security provided by classic-McEliece-348864 should be noted. These results have significant consequences for the choice and use of post-quantum cryptography algorithms in real-world applications, especially when key decapsulation speed and security are major considerations. Figure 15 and Figure 16 display identical results for encapsulation and decapsulation, respectively.

### 4.4. Result Comparison

In this study, we evaluated the performance of several variants of the BIKE and classic McEliece PQC algorithms for KEM in terms of key generation, encapsulation, and decapsulation operations on both Linux and Windows OSs. Table 9 shows the outcomes of the current study. Table 10 gives a condensed and collective comparison of the entire study’s findings. The evaluated algorithms variants include BIKE-L1 and BIKE-L3 and five variants of the classic McEliece scheme with different security levels, namely classic-McEliece-348864, classic-McEliece-460896, classic-McEliece-6688128, classic-McEliece-6960119, and classic-McEliece-8192128.

**Figure 17:**
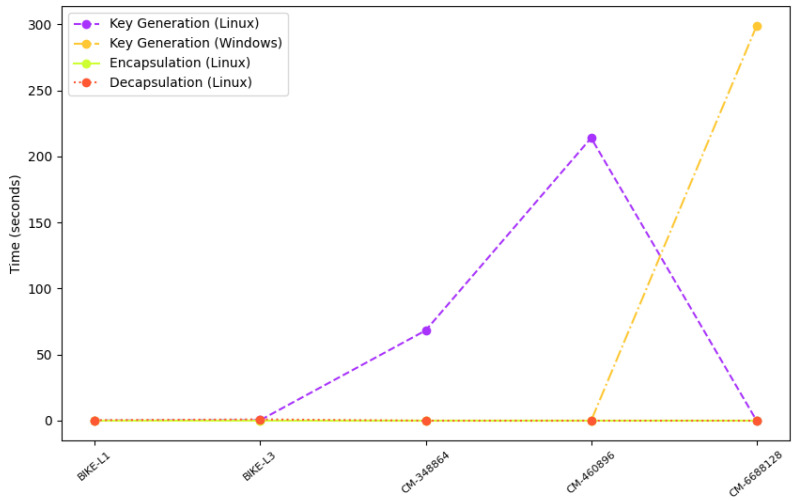
Comparison of results.

Figure 17 shows the experimental results indicating that classic-McEliece-348864 and classic-McEliece-460896 are the slowest algorithms in terms of key generation, taking 68.3 s and 214 s, respectively, on the Linux operating system. However, classic-McEliece-6688128 is the slowest algorithm on the Windows operating system, taking 298.8 s to generate a key. The BIKE-L1 and BIKE-L3 algorithms are significantly faster, taking 126.95 and 376.07 microseconds to generate a key, respectively, on the Linux operating system. The difference in the time required for key generation between the two BIKE algorithms is less than 0.25% on Linux.

Regarding encapsulation and decapsulation, BIKE-L1 is the fastest algorithm, taking 17.55 microseconds to encapsulate a message and 345.81 microseconds to decapsulate it, while BIKE-L3 is the slowest, taking 39.77 microseconds to encapsulate a message and 993.16 microseconds to decapsulate it. Classic-McEliece-348864 and classic-McEliece-460896 are faster than classic-McEliece-6688128 in terms of encapsulation and decapsulation on Linux, but on Windows, the performance of classic-McEliece-348864 and classic-McEliece-460896 is comparable to that of classic-McEliece-6688128. The graph results shown in Figure 5, Figure 6, Figure 7, Figure 8, Figure 9, Figure 10, Figure 11, Figure 12, Figure 13, Figure 14, Figure 15 and Figure 16 demonstrate the comparison of the algorithms variants based on their performance in terms of key generation, encapsulation, and decapsulation operations.

In conclusion, the results obtained from applying the proposed methodology to different PQC algorithms demonstrated its effectiveness in capturing their performance characteristics. The results showed that the performance of the PQC algorithms evaluated in this study vary significantly, depending on the type of operation, the security level, and the operating system used. Overall, BIKE-L1 and BIKE-L3 are the fastest algorithms, while classic-McEliece-348864 and classic-McEliece-460896 are the slowest algorithms, particularly in terms of key generation. Therefore, the choice of a PQC algorithm for KEM should be based on the specific requirements of the application, such as the desired level of security, the type of operation, and the platform.

Unlike the previous research, the results for the current study were obtained using the latest version of the liboqs v0.7.2. These results are based on all variants of two NIST forth-round finalists PQC algorithms, which belong to the same family. The experiments were performed on a standard laptop; specifically, we used a Lenovo IdeaPad 5i Pro 16AMD with an Intel® Core™ i5-11300H @ 3.11 GHz, NVIDIA® GeForce RTX™ 3050/3050 Ti Laptop GPU, and 16 GB RAM. We used Ubuntu 22.04.1 LTS and Windows 11 21H2 as the operating systems for the evaluation. We ensured that the experiments were conducted under controlled conditions to minimize external factors’ influence on the results.

## 5. Conclusions

With recent developments in quantum computing, conventional public cryptosystem become vulnerable, so post-quantum encryption algorithms should be investigated in the future. NIST has shortlisted several candidates, such as classic McEliece and BIKE, in this regard, which is yet to be standardized. This study presented the performance comparison of these two algorithms in terms of key generation, encapsulation, decapsulation, etc. The evaluation operation revealed valuable insights into the efficiency and practicality of these algorithms. The classic McEliece algorithm has an extremely large size for the public key, so it is possible that, in the future, it will not be a viable option. However, it functions at its highest level of efficiency when it is not required to frequently re-transmit the public key. In many implementations of the classic McEliece algorithm, the amount of memory required to store the extremely large key sizes is significantly higher. On Linux, the BIKE algorithm generates results that are noticeably superior. On Windows, however, the OQS liboqs does not support it by default. Both classic McEliece and BIKE will be supported by future TLS handshakes, and their respective levels of performance can be compared. The results of this research contribute to the ongoing efforts in standardizing post-quantum encryption algorithms and give vital recommendations for practitioners and researchers alike. When choosing an algorithm for secure communication, variables such as key size, performance requirements, and platform compatibility must be considered. As quantum computing advances, it is critical to monitor and adjust cryptographic systems to ensure the secrecy and integrity of sensitive data. differential power analysis (DPA) and differential fault analysis (DFA) attacks are a serious threat to the security of post-quantum cryptographic algorithms. By combining DPA and DFA attacks, attackers can extract secret information from cryptographic devices with greater ease. Further studies could focus on developing new countermeasures for side-channel attacks, including DPA and DFA attacks, by using techniques such as error detection and correction, fault-tolerant design, and side-channel masking. Future research might build on this work by investigating additional post-quantum encryption methods, evaluating their performance on various operating systems, and addressing real-world deployment circumstances. Ultimately, the effective development and implementation of post-quantum encryption algorithms will assure the long-term security of communications in the age of quantum computing, protecting sensitive data from new threats.

## Figures and Tables

**Table 1:** KEM and SIG algorithms—NIST PQC third-round finalists and alternate candidates.

Key Encapsulation Algorithms	Signature Algorithms
Classic McEliece	CRYSTALS-Dilithium
CRYSTALS-Kyber	Falcon
NTRU	Rainbow
Saber	
BIKE	GeMSS
HQC	SPHINCS+
FrodoKEM	Picnic
NTRU Prime	

**Table 2:** NIST standardized security levels.

Level	Description
1	At least as hard to break as AES128
2	At least as hard to break as SHA256
3	At least as hard to break as AES192
4	At least as hard to break as SHA384
5	At least as hard to break as AES256

**Table 3:** Summary of related work on PQC key exchange algorithms.

Ref.	Key Exchange Algorithm	NIST Security Level	Public Key Length (Bytes)	Private Key Length (Bytes)	Cipher Text Length (Bytes)	Key Gen	Encaps	Decaps	Key Exchange Mechanism
[21]	NewHope	×	×	×	×	✓	✓	✓	×
	Kyber	×	×	×	×	✓	✓	✓	×
	NTRU	×	×	×	×	✓	✓	✓	×
	Frodo	×	×	×	×	✓	✓	✓	×
[6]	Kyber-512	1	800	1632	736	✓	✓	✓	×
	NewHope-512-CCA	1	928	1888	1120	✓	✓	✓	×
	Kyber-768	3	1184	2400	1088	✓	✓	✓	×
	NTRU-HRSS-701	3	1138	1450	1138	✓	✓	✓	×
[22]	Kyber-512	1	×	×	×	×	×	×	✓
	Kyber-768	3	×	×	×	×	×	×	✓
	Kyber-1024	5	×	×	×	×	×	×	✓
	HQC-128	1	×	×	×	×	×	×	✓
	HQC-192	3	×	×	×	×	×	×	✓
	HQC-256	5	×	×	×	×	×	×	✓
	SIDH-p434	1	×	×	×	×	×	×	✓
	SIDH-p610	3	×	×	×	×	×	×	✓
	SIDH-p751	5	×	×	×	×	×	×	✓
[23]	Kyber	×	800	1632	736	✓	✓	✓	×
	NTRU	×	930	1234	930	✓	✓	✓	×
	NTRU	×	1138	1450	1138	✓	✓	✓	×
	Saber	×	672	1568	736	✓	✓	✓	×
	FrodoKEM	×	9616	19,888	9720	✓	✓	✓	×
	SIKE	×	330	374	346	✓	✓	✓	×
	SIKE	×	378	434	402	✓	✓	✓	×
	Kyber	×	378	434	402	✓	✓	✓	×
	NTRU	×	1230	1590	1230	✓	✓	✓	×
	Saber	×	992	2304	1088	✓	✓	✓	×
	FrodoKEM	×	15,632	31,296	15,744	✓	✓	✓	×
	NTRU Prime	×	1158	1763	1039	✓	✓	✓	×
	NTRU Prime	×	1039	1294	1167	✓	✓	✓	×
	SIKE	×	462	524	486	✓	✓	✓	×
	Kyber	×	1568	3068	1568	✓	✓	✓	×
	Saber	×	1312	3040	1472	✓	✓	✓	×
	SIKE	×	564	644	596	✓	✓	✓	×

**Table 4:** Related work on PQC signature algorithms.

Ref.	PQC Signature Algorithm	NIST Security Level	Public Key Length (Bytes)	Private Key Length (Bytes)	Signature Length (Bytes)	Sign	Verify
[6]	Dilithium	2	1472	3504	2701	✓	✓
	SPHINCS+ SHA256-128f	1	32	64	16,976	✓	✓
	Dilithium	3	1760	3856	3366	✓	✓
	SPHINCS+ SHA256-192f-	3	48	96	35,664	✓	✓
[22]	Falcon-512	1	×	×	×	✓	✓
	Falcon-1024	5	×	×	×	✓	✓
	Rainbow-I-Classic	1	×	×	×	✓	✓
	Rainbow-III-Classic	3	×	×	×	✓	✓
	Rainbow-V-Classic	5	×	×	×	✓	✓
	SPHINCS+-SHAKE256-128f-Robust	1	×	×	×	✓	✓
	SPHINCS+-SHAKE256-192f-Robust	3	×	×	×	✓	✓
	SPHINCS+-SHAKE256-256f-Robust	5	×	×	×	✓	✓
[23]	Dilithium	×	1184	2800	2044	×	×
	Falcon	×	1281	897	690	×	×
	Falcon	×	57,344	897	690	×	×
	Dilithium	×	1472	3504	2701	×	×
	Dilithium	×	1760	3856	3366	×	×
	Falcon	×	1793	2305	1330	×	×

**Table 5:** Algorithms to be standardized.

Key Encapsulation Algorithms	Signature Algorithms
CRYSTALS-Kyber	CRYSTALS-Dilithium
	Falcon
	SPHINCS+

**Table 6:** KEM algorithms from NIST PQC fourth-round finalists.

Key Encapsulation Algorithms
BIKE
Classic McEliece
HQC
SIKE

**Table 7:** Parameters of the classic McEliece algorithm.

PQC KEM Algo	Public Key Length (Bytes)	Private Key Length (Bytes)	Cipher Text Length (Bytes)	Plain Text Length (Byte)	Key Exchange Mechanism
Classic-McEliece-348864	261,120	6542	128	32	✓
Classic-McEliece-460896	524,160	13,568	188	32	✓
Classic-McEliece-6688128	1,044,992	13,892	240	32	✓
Classic-McEliece-6960119	1,047,319	13,908	226	32	✓
Classic-McEliece-8192128	1,357,824	14,080	240	32	✓

**Table 8:** Parameters of BIKE algorithm.

PQC KEM Algo	Public Key Length (Bytes)	Private Key Length (Bytes)	Cipher Text Length (Bytes)	Plain Text Length (Byte)	Key Exchange Mechanism
BIKE-L1	1541	5223	1573	32	✓
BIKE-L3	3083	10105	3115	32	✓

**Table 9:** Performance evaluation results of KEM PQC algorithm.

PQC KEM Algorithm	Iterations	Time (us): Mean	Pop. Stdev	CPU Cycles: Mean	Pop. Stdev	OS
**BIKE-L1**						
KeyGen	23,632	126.95	30.94	394,828	96,251	Linux
Encaps	170,951	17.55	1.59	54,534	4725	
Decaps	8676	345.81	9.34	1,075,522	29,006	
**BIKE-L3**						
KeyGen	7978	376.07	4.78	1,169,758	14,807	Linux
Encaps	74,536	39.77	0.68	123,643	1613	
Decaps	3021	993.16	10.58	3,089,290	32,866	
**Classic-McEliece-348864**						
KeyGen	44	68,329.50	9172.89	212,555,509	28,534,587	Linux
Encaps	299,954	10.002	11.93	31,053	37,102	
Decaps	90,678	33.08	21.42	102,840	66,622	
KeyGen	7	450,285.71	56,698.99	1,400,325,920	176,409,308	Windows
Encaps	2427	1236.09	668.84	3,844,480	1,668,744	
Decaps	3820	785.34	476.68	2,440,878	360,786	
**Classic-McEliece-460896**						
KeyGen	15	214,027.33	27,505.35	665,704,430	85,552,062	Linux
Encaps	172,298	17.37	4.27	54,097	13,203	
Decaps	36,954	81.18	4.62	252,414	14,341	
KeyGen	3	2,017,666.67	847,760.32	6,275,358,263	2,634,750,115	Windows
Encaps	1166	2574.61	1086.72	8,003,019	3,014,273	
Decaps	1160	2588.79	1016.61	8,056,956	2,910,747	
**Classic-McEliece-6688128**						
KeyGen	11	298,843.18	46,922.60	929,623,294	145,963,810	Linux
Encaps	97,774	30.68	4.43	95,344	13,702	
Decaps	28,488	105.31	9.29	327,501	28,858	
KeyGen	2	3,427,500.00	1,728,500.00	10,661,500,856	5,373,817,790	Windows
Encaps	796	3772.61	655.33	11,734,948	1,170,274	
Decaps	1315	2282.13	534.96	7,096,967	924,538	
**Classic-McEliece-6960119**						
KeyGen	10	313,892.00	71,613.75	976,436,744	222,772,222	Linux
Encaps	97,540	30.76	3.46	95,531	10,685	
Decaps	32,111	93.43	1.22	290,551	3477	
KeyGen	2	1,705,500.00	132,500.00	5,302,732,432	411,536,683	Windows
Encaps	755	3977.48	825.60	12,373,124	2,022,994	
Decaps	1308	2294.34	552.78	7,134,395	1,023,687	
**Classic-McEliece-8192128**						
KeyGen	9	349,939.22	91,937.66	1,088,568,757	285,994,218	Linux
Encaps	77,449	38.74	3.07	120,376	9427	
Decaps	28,877	103.89	2.34	323,066	7162	
KeyGen	1	3,099,000.00	0.00	9,635,977,370	0	Windows
Encaps	342	8792.40	2934.10	27,344,689	9,001,910	
Decaps	829	3620.02	1365.31	11,256,733	3,966,242	

**Table 10:** Result comparison of KEM PQC algorithm.

Algorithm	Key Generation (Linux)	Key Generation (Windows)	Encapsulation (Linux)	Decapsulation (Linux)
BIKE-L1	126.95 μs	-	17.55 μs	345.81 μs
BIKE-L3	376.07 μs	-	39.77 μs	993.16 μs
Classic-McEliece-348864	68.3 s	-	-	-
Classic-McEliece-460896	214 s	-	-	-
Classic-McEliece-6688128	-	298.8 s	-	-
Classic-McEliece-6960119	-	-	-	-
Classic-McEliece-8192128	-	-	-	-

## Data Availability

Not applicable.

## Abbreviations

AES	Advanced Encryption Standard
BIKE	Bit-flipping Key Encapsulation
CCA	Chosen Ciphertext Attack
DFA	Differential Fault Analysis
DH	Diffie–Hellman
DPA	Differential Power Analysis
EC	Elliptic Curve
ECC	Elliptic Curve Cryptography
ECDH	Elliptic Curve Discrete Logarithm
ECDSA	Elliptic Curve Digital Signature Algorithm
IEEE	Institute of Electrical and Electronics Engineers
IoT	Internet of Things
ISD	Information Set Decoding
KEM	Key Encapsulation Mechanism
LWC-SP	Lightweight Cryptography Standardization Process
NIST	National Institute of Standards and Technology
OQS	Open Quantum Safe
OS	Operating System
PQ	Post-Quantum
PQC	Post-Quantum Cryptography
RSA	Rivest–Shamir–Adleman
SCAs	Side-Channel Attacks
SHA-2	Secure Hashing Algorithm-2
SIG	Signatures
TLS	Transport Layer Security
